# Supplementary material for: Thromboembolic Events after COVID-19 Vaccination: An Italian Retrospective Real-World Safety Study
Source: Vaccines (Basel). 2023 Oct 10;11(10):1575. doi: 10.3390/vaccines11101575 (PMC10611339; doi:10.3390/vaccines11101575)
Supplement: Supplementary file 1 [file vaccines-11-01575-s001.zip › vaccines-2617984-supplementary.pdf]

Table S1. Events of COVID-19 vaccines reported in vascular cases of Campania Region from December 27th, 2020 to September 27th, 2022.

Adverse events	Oxford–AstraZeneca	Janssen	Moderna	Pfizer-BioNtech	Total
Headache	26	0	10	79	115
Hypertension	21	0	13	59	93
Fever	33	0	10	39	82
Hypotension	6	1	6	48	61
Tachycardia	15	3	6	32	56
Hot flashes	6	0	5	42	53
Blush	7	0	12	33	52
Nausea	4	0	6	30	40
Hypertensive crisis	6	0	3	29	38
Hematoma	11	2	5	20	38
Chills	12	0	3	22	37
Asthenia	6	1	5	23	35
Injection site pain	3	0	4	26	33
Fatigue	9	0	5	18	32
Arm pain	3	0	4	23	30
Itch	6	0	7	15	28
Arterial hypertension	5	0	3	15	23
Myalgia	5	0	2	14	21
Facial redness	4	0	0	17	21
Vomit	5	1	5	9	20
Diarrhea	2	0	4	12	18
Muscle pain	5	0	2	10	17
Paranesthesia	2	0	0	15	17
Thrombosis	8	1	2	6	17
Vertigo	3	1	4	9	17
Joint pain	6	0	0	10	16
Arthralgia	4	0	0	11	15
Dizziness	4	0	0	11	15
Dyspnoea	5	0	1	9	15
Migraine	3	0	4	8	15
General malaise	4	0	3	8	15
Syncope	2	1	5	5	13
Exhaustion	5	0	0	7	12
Orthostatic hypotension	1	5	3	2	11
Vasculitis	5	1	2	3	11
Bradycardia	1	0	4	5	10
Chills of cold	0	0	2	8	10
Chest pain	2	0	5	3	10
Generalized joint pain	3	0	2	5	10

Rash	0	0	3	7	10
Hyperpyrexia	4	0	3	3	10
Pre-syncope	0	5	3	2	10
Perspiration	1	0	0	9	10
Venous thrombosis	3	0	1	6	10
Chest pain	0	0	3	6	9
Generalized muscle pain	4	0	0	5	9
Cold feet	5	0	0	4	9
Blurred vision	4	0	0	4	8
Leg pain	3	0	0	5	8
Injection site edema	0	0	2	6	8
Pallor	1	0	2	5	8
Paresthesia of the limbs	1	1	2	4	8
Tremors	1	0	2	5	8
Arthromyalgia	4	0	0	3	7
Pain	3	0	2	2	7
Abdominal pain	4	1	0	2	7
Phlebitis	2	0	2	3	7
Malaise	0	0	0	7	7
Pyrexia	3	0	1	3	7
Gasp	2	0	3	2	7
Thrombophlebitis	1	1	1	4	7
Thrombophlebitis of the leg	4	0	0	3	7
Deep vein thrombosis	3	0	2	2	7
Acrocyanosis	2	0	1	3	6
Pulmonary embolism	1	0	4	1	6
Infarct	1	0	0	5	6
Livid	1	0	3	2	6
Urticaria	0	0	0	6	6
Thrombocytopenia	2	1	2	1	6
Sleepiness	2	0	1	3	6
Arrhythmia	0	0	2	3	5
Throat constriction	0	0	0	5	5
Abdominal cramps	1	0	0	4	5
Reddened skin	0	0	1	4	5
Increased fibrin D-dimer	2	0	1	2	5
Weakness	4	0	0	1	5
Back pain	2	0	2	1	5
Pain at the vaccination site	1	0	0	4	5
Epistaxis	1	0	1	3	5
Erythema	2	0	1	2	5
Feverish	0	0	0	5	5
Pressure fluctuation	0	0	1	4	5
Localized tingling	1	0	1	3	5

Swelling of the limb	0	0	3	2	5
Hyperemia	1	0	0	4	5
Headache with a feeling of heaviness	0	0	1	4	5
Paresthesia of lower limb	0	0	2	3	5
Loss of consciousness	0	0	0	5	5
Presincope	0	0	1	4	5
Feeling cold	3	0	1	1	5
Tremor	3	0	1	1	5
Thrombosis of the leg	2	0	0	3	5
Thrombosis of the saphenous vein	4	0	0	1	5
Hot flush	2	0	0	3	5
Hot flashes in the legs	2	0	0	3	5
Increased blood pressure	1	0	0	3	4
Broken capillary	3	0	0	1	4
Retching	0	0	1	3	4
Confusion	1	0	1	2	4
Elevated fibrin D-dimer	3	0	1	0	4
Difficulty breathing	0	0	0	4	4
Pain in one limb	1	0	1	2	4
Calf pain	1	0	1	2	4
Leg pain	0	0	1	3	4
Red-spotted rash	0	0	0	4	4
Cold ends	0	0	1	3	4
Tingling of feet/hands	2	0	0	2	4
Swelling in the limbs	2	0	0	2	4
Swelling of the feet	0	0	1	3	4
Lack of appetite	1	0	2	1	4
Hyposthenia	1	0	0	3	4
Ischemia	2	0	1	1	4
Lymphedema	0	0	1	3	4
Lymphadenopathy	1	0	0	3	4
Enlarged lymph nodes	0	0	0	4	4
Sore throat	1	0	1	2	4
Facial paresthesia	0	0	1	3	4
Blood loss	3	0	1	0	4
Petechiae	1	0	1	2	4
Labile blood pressure	2	1	0	1	4
Itching at the injection site	0	0	1	3	4
Bleeding	1	0	1	2	4
Confusion	1	0	0	3	4
Stunning	0	1	0	3	4
Excessive sweating	0	0	0	4	4
Tinnitus	0	1	1	2	4

Cough	0	0	1	3	4
Deep vein thrombosis of a limb	1	1	1	1	4
Blurred vision	1	0	1	2	4
Temperature increase	0	0	2	1	3
Boils	1	0	0	2	3
Burning in the leg	0	0	0	3	3
Persistent headache	2	0	0	1	3
Blood clot	3	0	0	0	3
Cramps	1	0	0	2	3
Vasovagal crisis	1	0	0	2	3
Diplopia	3	0	0	0	3
Capillary disorder	0	0	0	3	3
Pain in the lower extremities	2	0	0	1	3
Neck pain	0	0	0	3	3
Foot pain	2	0	0	1	3
Chest pain	1	0	1	1	3
Bone pain	0	0	1	2	3
Edema of the lower extremities	1	0	1	1	3
Hemorrhage	0	0	0	3	3
Increased fibrinogen	2	0	1	0	3
Tingling of the hand	1	0	1	1	3
Dizziness	2	0	0	1	3
Swelling of the face	0	0	0	3	3
Swelling of the tongue	1	0	2	0	3
Swelling of the legs	1	0	1	1	3
NAS swelling	0	0	2	1	3
Enlarged lymph nodes	1	0	1	1	3
Venous insufficiency	0	0	1	2	3
Localized numbness	0	0	0	3	3
Diastolic hypertension	2	0	0	1	3
Arterial hypotension	0	0	1	2	3
Hypothermia	1	0	1	1	3
Swollen lymph nodes	0	0	1	2	3
Stomach ache	1	0	0	2	3
Back pain	0	0	0	3	3
Cold hands and feet	1	0	0	2	3
Swollen eyes	1	0	0	2	3
Tightness in the chest	1	0	2	0	3
Paresthesia of the tongue	0	0	0	3	3
Paresthesia of the lips	1	0	1	1	3
Upper limb paresthesia	1	0	0	2	3
Semi-fainting	1	0	0	2	3
Feeling of warmth	1	0	1	1	3

Feeling cold in the limbs	0	0	0	3	3
Cold sweat	0	0	3	0	3
Faint	1	0	1	1	3
Thrombus	1	0	0	2	3
Deep vein thrombosis (limbs)	1	0	0	2	3
Varicosity	0	0	1	2	3
Diminished vision	1	0	0	2	3
Fatigue	1	0	0	1	2
Ageusia	0	0	0	2	2
Anemia	0	0	1	1	2
Angioedema	0	0	0	2	2
Anosmia	0	0	0	2	2
Anxiety	0	0	1	1	2
Sore joints	0	0	2	0	2
Swollen arm	0	0	2	0	2
Burning skin	1	0	0	1	2
Burning at the time of vaccination	0	0	0	2	2
Chest burning	0	0	1	1	2
Frontal headache	0	0	0	2	2
Cervicalgia	0	0	0	2	2
Cyanosis	0	0	0	2	2
Irregular menstrual cycle	0	0	0	2	2
Circulatory collapse	1	0	0	1	2
Hypotensive crisis	1	0	0	1	2
Dyspepsia	0	0	0	2	2
Speech disorder	1	0	0	1	2
Movement disorder	0	0	1	1	2
Speech disorder	1	0	0	1	2
Hearing disorder	1	0	0	1	2
Dysuria	0	0	0	2	2
Pain in one limb	0	0	1	1	2
Pain in the limbs	1	0	1	0	2
Armpit pain	0	0	1	1	2
Pain in the groin	0	0	0	2	2
Ear pain	1	0	0	1	2
Ankle pain	1	0	0	1	2
Leg pain	0	0	0	2	2
Pain in the hand	1	0	0	1	2
Widespread pain	0	0	1	1	2
Generalized pain	0	0	0	2	2
Intercostal pain	2	0	0	0	2
Retrosternal pain	1	0	1	0	2
Cardiac chest pain	1	0	1	0	2

Generalized pain	1	0	0	1	2
Ecchymosis	0	0	2	0	2
Eczema	0	0	2	0	2
Edema	0	0	1	1	2
Edema of the lips	2	0	0	0	2
Edema at the injection site	0	0	0	2	2
Hemiplegia	0	0	1	1	2
Cerebral hemorrhage	1	0	0	1	2
Enteritis	0	0	0	2	2
Epigastralgia	1	0	0	1	2
Spotted rash	0	0	0	2	2
Raynaud's phenomenon	0	0	0	2	2
Floppiness	0	0	0	2	2
Shortness of breath	0	0	1	1	2
Atrial fibrillation	1	0	0	1	2
Phlebitis of the lower limb	0	0	1	1	2
Abundant menstrual flow	0	0	0	2	2
End tingling	0	0	1	1	2
Capillary fragility	0	0	1	1	2
Swelling of the face	1	0	0	1	2
Swelling of the fingers	0	0	0	2	2
Bitter taste	0	0	0	2	2
Sores	0	0	0	2	2
Insomnia	0	0	0	2	2
Numbness of the limbs	0	0	2	0	2
Numbness of the lips	1	0	0	1	2
Hypoesthesia	0	0	0	2	2
Axillary lymphadenopathy	0	0	0	2	2
Skin spot	0	0	2	0	2
Stomach pain	1	0	1	0	2
Cold hands	0	0	1	1	2
Obnubilation	0	0	1	1	2
Arterial obstruction NAS	1	0	1	0	2
Palpitation	0	0	0	2	2
Palpitations	1	0	0	1	2
Arm paralysis	0	0	1	1	2
Bell's palsy	0	0	0	2	2
Hair loss	0	0	0	2	2
Weight loss	0	0	1	1	2
Pericarditis	0	0	1	1	2
Walnuts	0	0	1	1	2
Increased blood pressure	0	0	2	0	2
Low blood pressure	0	0	1	1	2
Increased protein C	1	0	1	0	2
Diffuse itching	0	0	0	2	2

Facial itching	0	0	0	2	2
Localized itching	0	0	1	1	2
Allergic reaction	1	0	0	1	2
Injection site reaction	1	0	0	1	2
Difficulty breathing	0	0	0	2	2
Shortness	2	0	0	0	2
Muscle stiffness	0	0	1	1	2
Retention	0	0	1	1	2
Ringing in your ears	0	0	1	1	2
Generalized redness	0	0	2	0	2
Sensation of warmth in the face	0	0	0	2	2
Feeling cold in the extremities	1	0	0	1	2
Feeling groggy	0	0	0	2	2
Sensation of hot flashes	2	0	0	0	2
Sense of oppression	2	0	0	0	2
Spasms	0	0	0	2	2
Increased sweating	0	0	0	2	2
Thrombocytopenia	2	0	0	0	2
Thromboembolism	0	1	0	1	2
Arterial thrombosis of a limb	1	0	0	1	2
Deep vein thrombosis of the leg	1	0	0	1	2
Deep femoral vein thrombosis	0	0	1	1	2
Swelling	0	0	0	2	2
Injection site swelling	0	0	0	2	2
Swelling at the vaccination site	0	0	0	2	2
Deep venous thrombosis (DVT)	1	0	0	1	2
Vasculitis of the legs	2	0	0	0	2
Peripheral vasoconstriction	0	0	0	2	2
Lowering the voice	0	0	0	1	1
Pressure lowering	0	0	1	0	1
Lowering blood pressure	0	0	1	0	1
Cardiac acceleration	0	0	1	0	1
Aphasia	0	0	0	1	1
Aphtha	0	0	0	1	1
Psychomotor agitation	0	0	0	1	1
Allergy	1	0	0	0	1
Olfactory hallucination	1	0	0	0	1
Hallucinations	0	0	0	1	1
Skin discoloration	0	0	1	0	1
Altered erection	0	0	0	1	1

Alteration of smell	1	0	0	0	1
Altered attention	0	0	1	0	1
Amenorrhea	1	0	0	0	1
Transient amnesia	1	0	0	0	1
Anaphylaxis	0	0	0	1	1
Antithrombin III increased	1	0	0	0	1
Reduced appetite	1	0	0	0	1
Extended APTT	1	0	0	0	1
Cardiac arrest	0	0	0	1	1
Reddening	0	0	0	1	1
Skin redness	0	0	0	1	1
Redness of the face	0	0	0	1	1
Redness at the injection site	0	0	0	1	1
Septic arthritis	0	0	1	0	1
Abscess at the injection site	0	0	0	1	1
Ascites	0	0	0	1	1
Pale appearance	0	0	0	1	1
Atherosclerosis of the aorta	0	0	0	1	1
Atony	0	0	0	1	1
Panic attacks	0	0	1	0	1
Sweating attack	0	0	0	1	1
Transient ischemic attack	0	0	0	1	1
Abnormal weight gain	0	0	0	1	1
Increased blood basophils	1	0	0	0	1
Added beats	1	0	0	0	1
Device lock	0	0	1	0	1
Dry mouth	0	0	1	0	1
Chills of fever	0	0	0	1	1
Burning eyes	1	0	0	0	1
Breast burning	0	0	1	0	1
Fall	0	0	0	1	1
Falls	0	0	0	1	1
Heat at the injection site	0	0	0	1	1
Hypertensive heart disease	0	0	0	1	1
Swollen ankles	0	0	1	0	1
Exertional headache	1	0	0	0	1
NAS headache	0	0	0	1	1
Occipital headache	0	0	0	1	1
Cephalgia	0	0	0	1	1
Red patches	1	0	0	0	1
Cystitis	0	0	0	1	1
Claudication	0	0	0	1	1
Reduced HDL cholesterol	1	0	0	0	1
Abdominal colic	1	0	0	0	1
Confabulation	0	0	0	1	1

Conjunctivitis	0	0	1	0	1
Decreased platelet count	1	0	0	0	1
Muscle contraction	0	0	1	0	1
Increased uterine contractions	0	0	0	1	1
Tightening to the chest	1	0	0	0	1
Constriction of the chest	0	0	0	1	1
Chest constriction	0	0	0	1	1
Leg cramps	0	0	0	1	1
Cramps of the lower extremities	1	0	0	0	1
Foot cramps	0	0	0	1	1
Intestinal cramps	1	0	0	0	1
Absence crisis	0	0	0	1	1
Epileptic seizure	0	0	0	1	1
Rough skin	0	0	1	0	1
Dry skin	0	0	0	1	1
Abnormal fibrin D-dimer	1	0	0	0	1
Weakness of the upper extremities	0	0	0	1	1
Muscle weakness	0	0	0	1	1
Death from natural causes	0	0	1	0	1
Motor deficit	0	0	1	0	1
Difficult swallowing	0	0	0	1	1
Painful swallowing	0	0	0	1	1
Delirium	1	0	0	0	1
Depression	0	0	0	1	1
Dermatomyositis	0	0	0	1	1
Head and gaze deviation	0	0	0	1	1
Diabetes	0	0	0	1	1
Watery diarrhea	1	0	0	0	1
Blood diarrhea	1	0	0	0	1
Difficulty walking	0	0	0	1	1
Difficulty sleeping	0	0	0	1	1
Difficulty walking	0	0	0	1	1
Difficulty chewing	0	0	1	0	1
Inferior vena cava dilatation	0	0	0	1	1
Dysarthria	0	0	0	1	1
Dysesthesia of a limb	1	0	0	0	1
Dehydration	0	0	1	0	1
Disorientation	0	0	0	1	1
Peripheral artery dissection	0	0	0	1	1
Visual disturbances	1	0	0	0	1
Heart disorder	1	0	0	0	1
Eye disorder	0	0	0	1	1

Bleeding disorder	0	0	0	1	1
Swallowing disorder	0	0	0	1	1
Menstrual disorder	0	0	0	1	1
Visual disturbance	0	0	0	1	1
Stiff fingers	0	0	1	0	1
Decreased diuresis	0	0	0	1	1
Tooth pain	0	0	0	1	1
Pain in the head	1	0	0	0	1
Scalp pain	0	0	0	1	1
Pain in the side	0	0	1	0	1
Hip pain	0	0	0	1	1
Thigh pain	0	0	0	1	1
Pain in the face	0	0	0	1	1
Throat pain	0	0	1	0	1
Buttock pain	1	0	0	0	1
Ankle pain	0	0	0	1	1
Stomach pain	0	0	0	1	1
Joint pain at the injection site	0	1	0	0	1
Joint pain at the vaccination site	0	0	0	1	1
Axillary pain	0	0	0	1	1
Cervical pain	0	0	0	1	1
Stomach pain	1	0	0	0	1
Back pain	1	0	0	0	1
Pain during injection	0	0	1	0	1
Epigastric pain	0	0	0	1	1
Administration site pain	0	0	0	1	1
Stabbing pain	0	0	0	1	1
Pain	0	0	0	1	1
Musculoskeletal pain	1	0	0	0	1
Osteoarticular pain	0	0	0	1	1
Precordial pain	0	0	0	1	1
Renal pain	1	0	0	0	1
Dull pain in the head	1	0	0	0	1
Burning pain	0	0	0	1	1
Vascular pain	1	0	0	0	1
Arthritic pain	0	0	0	1	1
Capillary ectasia	0	0	0	1	1
Edema in the neck	0	0	0	1	1
Edema of the limbs	1	0	0	0	1
Edema of the lip	0	0	0	1	1
Edema of the face	0	0	0	1	1
Edema of the upper limb	1	0	0	0	1
Ankle edema	1	0	0	0	1
Edema of the glottis	0	0	0	1	1

Edema of the throat	0	0	0	1	1
Edema of the fingers of the hands	0	0	0	1	1
Edema of a lower limb	0	0	1	0	1
Ocular edema	0	0	1	0	1
Venous edema	0	0	0	1	1
Hematuria	0	0	0	1	1
Hemiparesthesia	1	0	0	0	1
Conjunctival hemorrhage	1	0	0	0	1
Eye haemorrhage	1	0	0	0	1
Subconjunctival hemorrhage	0	0	0	1	1
Increased blood eosinophils	0	0	1	0	1
Hypertensive episodes	0	0	0	1	1
Episode of loss of consciousness	1	0	0	0	1
Hypertensive episode	0	0	0	1	1
Skin erythema	0	0	0	1	1
Palmar erythema	1	0	0	0	1
Bullous rash	0	0	1	0	1
Facial rash	0	0	0	1	1
Generalized rash	0	0	0	1	1
Rash on the trunk	0	0	0	1	1
Pimple-type rash	0	0	0	1	1
Exacerbation of disease	0	0	0	1	1
Exanthema	0	0	0	1	1
Ventricular extrasystole	0	0	0	1	1
Extrasystoles	0	0	0	1	1
Reddened face	0	0	0	1	1
Hunger	1	0	0	0	1
Annoyance	0	0	0	1	1
Discomfort in the head	0	0	0	1	1
Discomfort in the abdomen	0	0	1	0	1
Oral discomfort	0	0	0	1	1
Respiratory fatigue	1	0	0	0	1
Mild fever	0	0	1	0	1
Fatty liver	0	0	0	1	1
Fibrillation	0	0	0	1	1
Fibromyalgia	0	0	0	1	1
Ringing in the ears	0	0	1	0	1
Phlebitis of the arm	1	0	0	0	1
Phlebolinfedema	0	0	0	1	1
Phlebothrombosis	0	0	0	1	1
Phlebothrombosis of the lower extremities	0	0	0	1	1
Tingling	0	0	0	1	1

Tingling of the tongue	1	0	0	0	1
Tingling of the lips	0	0	0	1	1
Photophobia	0	0	0	1	1
Photopsy	1	0	0	0	1
Vascular fragility	0	0	0	1	1
Localized coldness	0	0	1	0	1
Increased heart rate	1	0	0	0	1
Low heart rate	0	0	0	1	1
Frequency of urination	0	0	0	1	1
Abnormal cognitive function	1	0	0	0	1
Gingivitis	0	0	0	1	1
Glossitis	0	0	0	1	1
Abdominal bloating	1	0	0	0	1
Joint swelling at the vaccination site	0	0	1	0	1
Swelling of the face	0	0	0	1	1
Swelling of the hands	1	0	0	0	1
Metallic taste	0	0	0	1	1
Herpes NAS	0	0	0	1	1
Ocular herpes	1	0	0	0	1
Ischemic stroke	0	0	0	1	1
Unable to swallow	0	0	0	1	1
Urinary incontinence	0	0	0	1	1
Joint soreness	0	0	1	0	1
Injection site hardening	0	0	1	0	1
Spleen infarction	0	0	0	1	1
Intestinal infarction	0	0	0	1	1
Myocardial infarction	0	0	0	1	1
Gastrointestinal infection	1	0	0	0	1
Inflammation	0	0	0	1	1
Inflammation of the mucous membrane	0	0	0	1	1
Inflammation of the stomach	0	0	0	1	1
Injection site inflammation	1	0	0	0	1
Lymph node inflammation	0	0	0	1	1
Ocular inflammation	0	0	0	1	1
Instability	1	0	0	0	1
Lymphatic insufficiency	0	0	1	0	1
Mitral regurgitation	0	0	0	1	1
Renal	1	0	0	0	1
Acute renal failure	0	0	1	0	1
Acute on chronic renal failure	0	0	1	0	1
Chronic venous insufficiency	0	0	0	1	1
Peripheral venous insufficiency	0	0	0	1	1
Interleukin 6 increased	1	0	0	0	1

Numbness	1	0	0	0	1
Tongue numbness	0	0	0	1	1
Azotemia	1	0	0	0	1
Hypercholesterolemia	0	0	0	1	1
Hyperesthesia	0	0	0	1	1
Hyperhomocysteinemia	0	0	0	1	1
Hypersalivation	0	0	0	1	1
Hypersomnia	0	0	0	1	1
Aggravated hypertension	0	0	0	1	1
NAS hypertension	0	0	0	1	1
Venous hypertension	0	0	0	1	1
Hypertensive	0	0	0	1	1
Hypertransaminasemia	0	0	0	1	1
Hyperuricaemia	1	0	0	0	1
Hypoglycaemia	0	0	0	1	1
Hyponatremia	0	0	0	1	1
Cerebral ischemia	1	0	0	0	1
Chronic cerebral ischemia	0	0	0	1	1
Ischemia NAS	1	0	0	0	1
Splenic ischemia	0	0	0	1	1
Blue lips	1	0	0	0	1
Swollen lips	0	0	0	1	1
Labyrinthitis	0	0	0	1	1
Lachrymation	0	0	0	1	1
Plaintive	0	0	1	0	1
Acute myeloid leukemia	1	0	0	0	1
Decreased libido	0	0	0	1	1
Joint limitation	0	0	0	1	1
NAS Lymphedema	0	0	0	1	1
Generalized lymphadenopathy	0	0	1	0	1
Submandibular lymphadenopathy	0	0	0	1	1
Enlarged axillary lymph nodes	0	0	0	1	1
Inguinal lymph nodes	0	0	0	1	1
Lymphostasis	0	0	0	1	1
White tongue	0	0	1	0	1
Lividura	0	0	0	1	1
Lumbago	0	0	0	1	1
Lupus positive anticoagulant	0	0	0	1	1
Reddened hands	0	0	0	1	1
Difficult focusing	0	0	1	0	1
Microembolism	0	0	0	1	1
Difficult urination	0	0	0	1	1

Myocarditis	0	0	0	1	1
Myodesopsia	0	0	1	0	1
Decreased mobility	0	0	0	1	1
Nephritis	0	0	0	1	1
NAS neuropathy	0	0	0	1	1
Peripheral neuropathy	0	0	0	1	1
Subcutaneous nodule	1	0	0	0	1
Painful red eyes	0	0	0	1	1
Cerebral artery occlusion	0	0	0	1	1
Corneal blurring	0	0	1	0	1
Oligoanuria	0	0	0	1	1
Oliguria	0	0	0	1	1
Chest tightness	1	0	0	0	1
Chest tightness	0	0	0	1	1
Stye	1	0	0	0	1
Dulling	0	0	0	1	1
Autoimmune pancreatitis	0	0	1	0	1
Subacute pancreatitis	0	0	1	0	1
Panniculitis	0	0	0	1	1
Right side paralysis	1	0	0	0	1
Paralysis of the tongue	0	0	1	0	1
Pseudobulbar paralysis	0	0	0	1	1
Paresthesia of the mouth	0	0	1	0	1
Pathology of teeth	1	0	0	0	1
Pathology of the jaw	1	0	0	0	1
Loss	0	0	0	1	1
Loss of taste	0	0	0	1	1
Loss of balance	0	0	1	0	1
Loss of smell	0	0	0	1	1
Memory loss	0	0	0	1	1
Vision loss	1	0	0	0	1
Unspecified vision loss	0	0	0	1	1
Transient loss of consciousness	0	0	1	0	1
Menstrual losses	1	0	0	0	1
Vaginal discharge	1	0	0	0	1
Acute pericarditis	0	0	0	1	1
Heaviness in the limbs	0	0	0	1	1
Heaviness of the limbs	1	0	0	0	1
Heaviness of the leg	0	0	0	1	1
Burning feet	0	0	0	1	1
Pyrexia of unknown origin	1	0	0	0	1
Pinching of the skin	0	0	0	1	1
Generalized tingling	0	0	1	0	1
Pollachiuria	0	0	0	1	1

Swollen wrists	0	0	0	1	1
Knows	0	0	0	1	1
Purple	0	0	0	1	1
Head pressure	1	0	0	0	1
Increased eye pressure	1	0	0	0	1
Balance problem	0	0	1	0	1
Prominence of superficial vein	1	0	0	0	1
Abnormal C-reactive protein	1	0	0	0	1
Proteinuria	0	0	0	1	1
Itching - generalized	0	0	1	0	1
Itchy eyes	0	0	0	1	1
Itchy eye	0	0	0	1	1
Skin itching	0	0	1	0	1
Generalized itching	0	0	0	1	1
Oral itching	0	0	0	1	1
Rachialgia	0	0	0	1	1
Hoarseness	0	0	0	1	1
Anaphylactoid reaction	0	0	0	1	1
Reaction at the vaccination site	0	0	0	1	1
Vagal reaction	0	0	0	1	1
Vasovagal reaction	0	0	0	1	1
Tricuspid regurgitation	0	0	1	0	1
Regurgitation of the pulmonary valve	0	0	1	0	1
Vasomotor rhinitis	0	0	0	1	1
Urinary retention	0	0	0	1	1
Rupture of blood vessel in the eye	0	0	0	1	1
Nosebleeds	0	0	1	0	1
Blood in the urine	1	0	0	0	1
Bleeding from the nose	0	0	0	1	1
Low oxygen saturation	0	0	1	0	1
Sciatica	0	0	0	1	1
Dry mouth	0	0	0	1	1
Secretion	0	0	1	0	1
Burning sensation in the limbs	0	0	0	1	1
Burning sensation of the oral mucosa	1	0	0	0	1
Sensation of warmth in the limbs	0	0	0	1	1
Dizziness feeling	0	0	0	1	1
Feeling of constriction	0	0	0	1	1
Sensation of electroshock	0	0	1	0	1

Tingling sensation	1	0	0	0	1
Feeling cold in the lower extremities	1	0	0	0	1
Feeling cold on the skin	1	0	0	0	1
Sensation of chest tightness	1	0	0	0	1
Choking sensation	0	0	0	1	1
Feeling of foggy head	0	0	0	1	1
Localized sensation of heat	0	0	0	1	1
Skin rash	0	0	0	1	1
Anaphylactic shock	0	0	0	1	1
Hypovolemic shock	0	0	1	0	1
Vasovagal syncope	0	0	0	1	1
Raynaud's syndrome	1	0	0	0	1
DRESS syndrome	0	0	1	0	1
Vertiginous syndrome	0	0	1	0	1
Hemorrhagic suffusion	0	0	0	1	1
Slumber	0	0	0	1	1
Abdominal spasm	0	0	0	1	1
Anxious state	0	0	0	1	1
State of agitation	0	0	0	1	1
Artery stenosis	0	0	0	1	1
Constipation	0	0	0	1	1
Stomatitis	1	0	0	0	1
Mental numbness	1	0	0	0	1
Sweats	1	0	0	0	1
Sweats	1	0	0	0	1
Tachypnea	0	0	0	1	1
Tension of the mandibular muscles	0	0	0	1	1
Confused head	0	0	0	1	1
Muffled head	0	0	1	0	1
Button head	0	0	0	1	1
Tic	0	0	0	1	1
Thoracoalgia	0	0	1	0	1
Hack	0	0	0	1	1
Increased transaminases	0	0	1	0	1
Finger tremor	0	0	0	1	1
High triglycerides	1	0	0	0	1
Thromboembolism of the lung	0	0	1	0	1
Venous thromboembolism	0	0	0	1	1
Deep thrombophlebitis	1	0	0	0	1
Thrombolysis	0	0	1	0	1
Thrombosis of the arm	1	0	0	0	1
Femoral artery thrombosis	0	0	1	0	1
Axillary vein thrombosis	0	0	0	1	1

Thrombosis of varicose veins	1	0	0	0	1
Venous thrombosis (limbs)	1	0	0	0	1
Venous thrombosis of the arm	1	0	0	0	1
Deep vein thrombosis of the arm	0	0	0	1	1
Left deep vein thrombosis	0	0	1	0	1
Auricular swelling	0	1	0	0	1
Attenuated hearing in both ears	0	1	0	0	1
Dark urine	0	0	0	1	1
Flushes in the face	0	0	1	0	1
Hot flashes in the face	0	0	0	1	1
Change in blood pressure	1	0	0	0	1
Bleeding esophageal varices	0	0	0	1	1
Cryoglobulinemic vasculitis	0	0	0	1	1
Vasculitis of the fingers	0	0	0	1	1
Diffuse vasculitis	0	0	0	1	1
NAS Vasculitis	0	0	0	1	1
Vasoconstriction	0	0	0	1	1
Peripheral vasodilation	0	0	0	1	1
Relaxed vein	1	0	0	0	1
Varicose vein	0	0	1	0	1
Dilated veins	0	0	0	1	1
Varicose veins	0	0	1	0	1
Joint effusion	0	0	0	1	1
Blurred vision	1	0	0	0	1
Low vitamin D	1	0	0	0	1
Xerostomia	0	0	0	1	1
Total	564	34	385	1468	2451
